# Cellular adaptations of fission yeast to inositol starvation

**DOI:** 10.1128/mbio.01864-26

**Published:** 2026-09-21

**Authors:** Ana M. Sanchez, Jill Babor, Michelle Saoi, Isabel Prucker, Henning J. Jessen, Beate Schwer, Stewart Shuman

**Affiliations:** 1 Molecular Biology Program, Memorial Sloan Kettering Cancer Center 5803, New York, New York, USA; 2 Cell Metabolism Core Facility, Memorial Sloan Kettering Cancer Center 5803, New York, New York, USA; 3 Institute of Organic Chemistry, University of Freiburg 9174, Freiburg, Germany; 4 Department of Microbiology and Immunology, Weill Cornell Medical College 12295, New York, New York, USA; Georgia Institute of Technology, Atlanta, Georgia, USA

**Keywords:** inositol starvation, gene expression, inositol pyrophosphates, inorganic polyphosphate

## Abstract

Inositol is an essential nutrient and a precursor of phosphorylated metabolites that play important roles in cell physiology. Here we characterize the impact of intracellular inositol deficiency, taking advantage of fission yeast *Schizosaccharomyces pombe*, an obligate inositol auxotroph, as a model system. We find that cells deprived of inositol ceased growth after two or three rounds of division, with genome content indicative of a G2 arrest. Inositol starvation elicited (i) widespread changes in the expression of genes involved in lipid metabolism and inositol polyphosphate dynamics, associated with an across-the-board elevation of triacylglycerol lipids; (ii) depletion of glycerophosphoinositol and accumulation of glycerophosphocholine and choline; (iii) selective depletion of inositol pyrophosphate 1,5-IP_8_, and depletion of polyphosphate; (iv) upregulation of autophagy genes; (v) downregulation of genes for the biogenesis and function of ribosomes and tRNAs, accompanied by conversion of polysomes to 80S monosomes; and (vi) large-magnitude changes in mRNAs encoding transcription factors. Our study underscores distinctive and shared features of the cellular responses to inositol deficiency versus phosphate deficiency that enhance our understanding of nutrient stress.

**IMPORTANCE:**

Inositol is an essential precursor of diverse phosphorylated derivatives that govern cell signaling, glucose and lipid metabolism, and cellular phosphate homeostasis. To probe how cells adapt to inositol deprivation, we exploited the fission yeast *Schizosaccharomyces pombe*, which is unable to synthesize inositol and requires exogenous inositol for growth. Via genome-wide analysis of changes in mRNA levels and tracking the status of inositol-adjacent metabolites, we highlight adaptive responses specific to inositol starvation that affect lipid metabolism and inositol pyrophosphate signaling. Clues to the origins of the transcriptome remodeling during inositol starvation emerged from the large-magnitude increases in mRNAs encoding certain RNA polymerase II transcription factors, which correlated with increased expression of their putative client genes.

## INTRODUCTION

**Fig 1:**
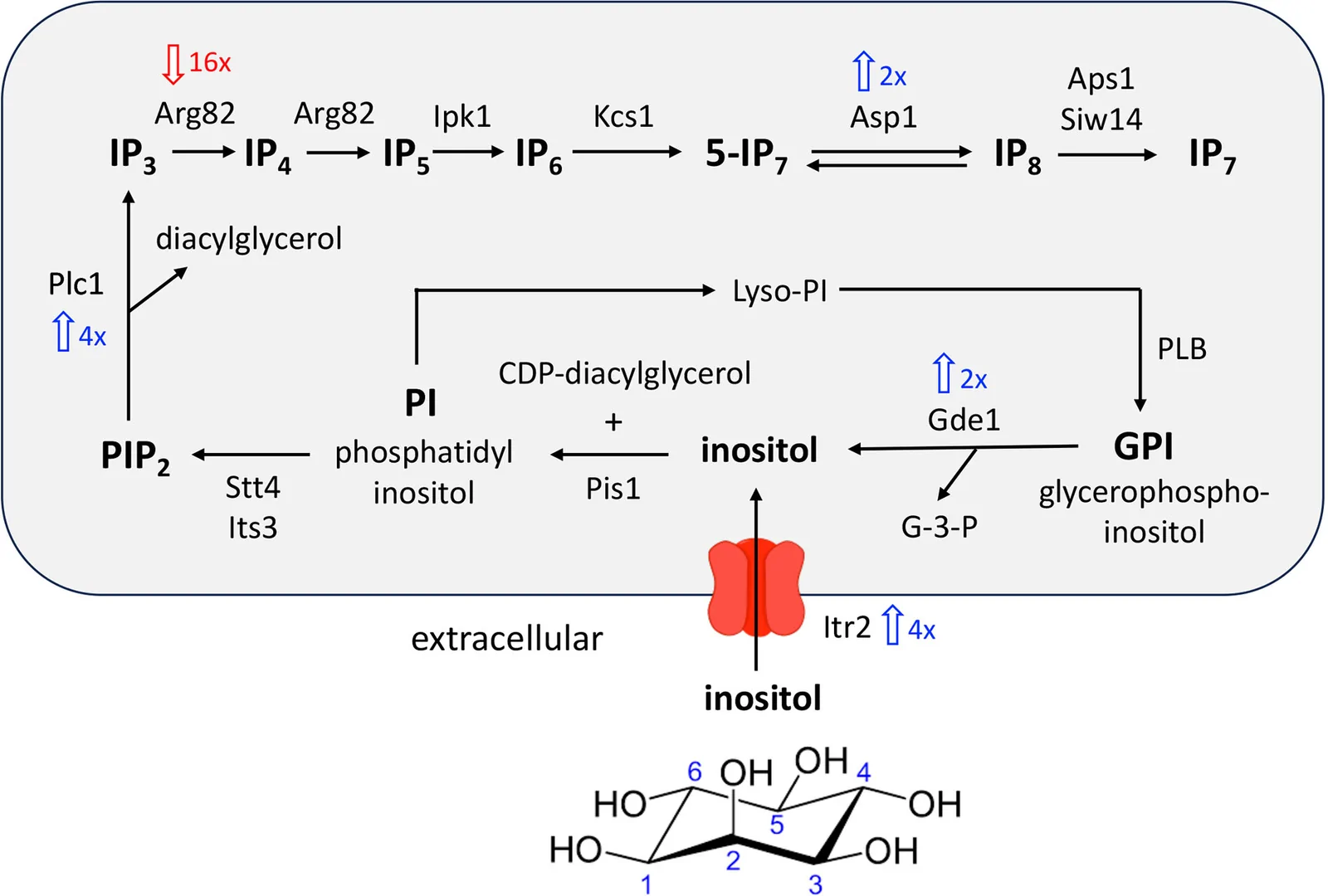
Inositol metabolism in fission yeast. Schematic illustrating several intracellular fates of *myo*-inositol (chemical structure shown with carbon atoms numbered) after Itr2-mediated import across the plasma membrane. Fold changes, up or down, in the levels of mRNAs encoding Itr2 and the indicated enzymes of inositol phosphate metabolism in inositol-starved cells versus inositol-replete cells are indicated by vertical arrows.

*Myo*-inositol (Fig. 1; hereafter inositol) is an essential metabolite obtained by cells from their environment or via *de novo* synthesis from glucose-6-phosphate (G6P). Inositol is a precursor of diverse phosphorylated derivatives—glycerophosphoinositol (GPI), inositol polyphosphates, inositol pyrophosphates, and phosphoinositide lipids—with distinct and important functions in cell physiology. These include roles as second messengers, regulators of glucose and lipid metabolism, and effectors of phosphate homeostasis. Inositol is “generally recognized as safe” by the FDA, and a variety of health benefits have been imputed to taking inositol as a dietary supplement, e.g., reducing risk of metabolic syndrome, improving insulin action, reducing risk of gestational diabetes, lowering cholesterol, and ameliorating polyendocrine metabolic ovarian syndrome. Depletion of intracellular inositol in the CNS is thought to underlie the efficacy of lithium and valproate in treating bipolar disorder (1).

Cellular inositol homeostasis has been investigated in budding yeast *Saccharomyces cerevisiae*, an organism with the capacity to synthesize inositol from G6P via the inositol-3-phosphate synthase enzyme Ino1, followed by dephosphorylation of inositol-3-phosphate to inositol (reviewed in reference 2). Budding yeast *ino1* mutants have been deployed to interrogate the effects of inositol deprivation (2). The fission yeast *Schizosaccharomyces pombe* lacks Ino1 and is an obligate inositol auxotroph that can be restored to inositol prototrophy by expression of *Pichia pastoris* Ino1 (3). Exogenous inositol in the fission yeast growth medium is imported into the cell by plasma membrane transporter Itr2, which is essential for fission yeast growth (4) (Fig. 1). Thus, fission yeast provides an excellent model for the analysis of how wild-type eukaryal cells adapt to inositol starvation, especially given early findings that fission yeast are more resistant to loss of viability during inositol deprivation than are artificially engineered budding yeast *ino1* mutant inositol auxotrophs (5). Several studies have documented the effects of fission yeast inositol starvation on phospholipid synthesis, unfolded protein response, viability, and apoptotic cell death (5–7). However, there has not been a systematic analysis of fission yeast inositol starvation, with particular attention to effects on gene expression, comparable to the recent studies of fission yeast adaptive responses to phosphate starvation (8–10). Here, we present a time-resolved study of inositol starvation of fission yeast, which highlights both distinctive and shared features *vis-à-vis* the stresses incurred during phosphate starvation.

## RESULTS

### Acute inositol starvation triggers growth arrest and loss of viability

**Fig 2:**
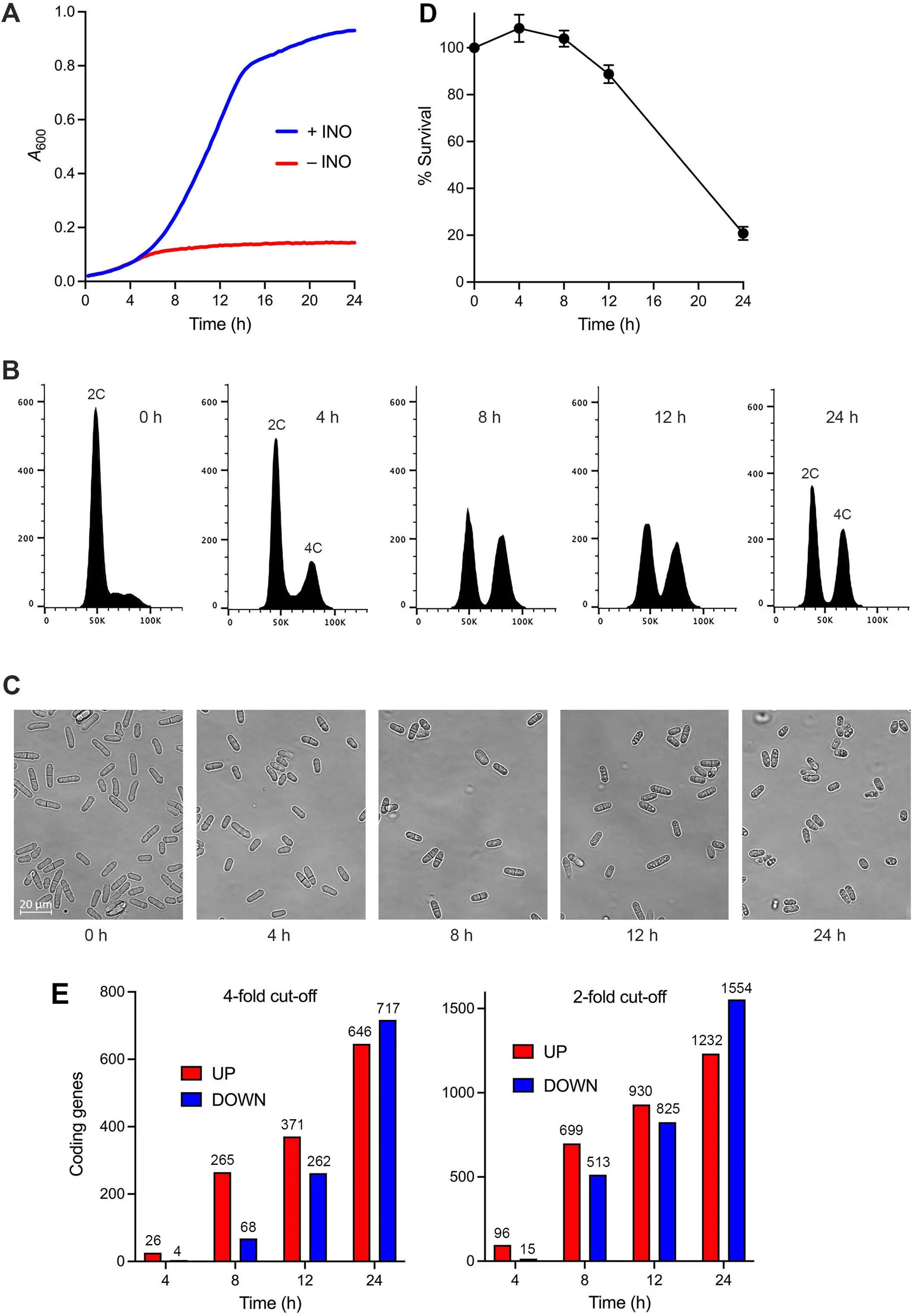
*S. pombe* response to inositol starvation. (**A**) Cells were grown in ePMGT containing 55 µM inositol ePMGT(+INO) to an *A*_600_ of ~0.6. Cells were harvested, washed, and resuspended at an *A*_600_ of 0.1 in either ePMGT(+INO) or ePMGT(–INO). Aliquots (100 µL) were transferred to a 96-well plate, and growth was monitored over 24 h using an Agilent BioTek Epoch 2 plate reader, taking *A*_600_ readings every 15 min. The growth curves shown are the average of three biological replicates (independent cultures). (**B**) DNA content of cells was measured by flow cytometry (11) before (0 h) and 4, 8, 12, and 24 h after transfer to inositol-free medium. Histograms of DNA content measured by flow cytometry are shown. The x- and y-axes indicate fluorescence intensity and cell number, respectively. (**C**) Cells sampled prior to (0 h) and at sequential times after transfer to –INO medium were visualized by brightfield microscopy (630× magnification). (**D**) Survival of inositol-starved fission yeast cells. Aliquots of cells from liquid cultures were withdrawn prior to (time 0) and 4, 8, 12, and 24 h after transfer to –INO medium, and cell counts were determined using a hemacytometer. Serial dilutions were plated on inositol-containing ePMGT agar and incubated at 30°C for 3 days. Viable colony counts were normalized to the time 0 h control. Percent survival is plotted as a function of starvation time. Each datum is the average of three or four biological replicates ± SEM. (**E**) Transcriptional response to inositol starvation. RNA-seq was performed on poly(A)^+^ RNA isolated from wild-type cells before (0 h) and after 4, 8, 12, and 24 h of starvation. Three biological replicates were sequenced for each time point. Differential expression analysis was performed using the following criteria: a fold change cutoff of ±4 (left panel) or ±2 (right panel) in normalized transcript read level compared to the 0 h time point, and an adjusted *P* value of ≤0.05. Bar graphs show coding genes that were upregulated or downregulated at the specified times following transfer to inositol-free medium.

We tracked growth by measuring *A*_600_ of triplicate cultures after transfer from rich synthetic ePMGT medium containing 55 µM inositol to medium lacking inositol. *S. pombe* grew logarithmically for 14 h in +INO medium before transitioning to stationary phase (Fig. 2A). By contrast, cells starved for inositol grew at a near-normal rate for 4 h in –INO medium before arresting growth by 6–8 h (Fig. 2A).

Light microscopy and flow cytometry analyses were performed on fission yeast cells prior to (time 0) and after 4, 8, 12, and 24 h of inositol starvation. The time 0 cells showed rod-shaped morphology (Fig. 2C) and an asynchronously cycling population with a majority 2C and minority 4C DNA content (Fig. 2B). The 4C cell population increased steadily at 4 and 8 h post-starvation, concomitant with a decrement in the 2C population; the flow cytometry profile remained virtually static at 12 and 24 h (Fig. 2B). We surmise that inositol-starved cells entered into an arrested state with a slightly smaller size at 24 h post-starvation (Fig. 2C).

To gauge survival, cells were collected prior to (time 0) and after 4, 8, 12, and 24 h of inositol starvation, counted microscopically, and then plated on ePMGT(+INO) agar medium. Viable colony counts were normalized to the time 0 control. We found that cells remained viable for 12 h in –INO medium before suffering a decline in viability (to 20% survival) between 12 and 24 h of inositol starvation (Fig. 2D). Fission yeast cells are apparently more sensitive to inositol starvation than to phosphate or nitrogen starvation, albeit less sensitive to glucose starvation. Phosphate starvation elicits G0 quiescence with no loss of viability after 2 days of phosphate starvation, followed by a gradual decrement to 44% survival after 14 days and 4% survival after 28 days (9). Cells starved for nitrogen arrest in G0 and remain fully viable for up to 28 days (12). By contrast, ≤1% of fission yeast cells are viable 1 day after a switch from rich medium to medium lacking glucose (13).

### Transcriptional profiling of inositol-starved fission yeast

We performed RNA-seq on poly(A)^+^ RNA isolated from fission yeast cells (three biological replicates) prior to (time 0) and 4, 8, 12, and 24 h after transfer to –INO medium. We found that the number of ±2-fold dysregulated protein-coding genes increased sharply between 4 h (*n* = 111) and 8 h (*n* = 1,212) and continued to rise gradually at 12 h (*n* = 1,755) and 24 h (*n* = 2,786) (Fig. 2E, right panel). It was noteworthy that upregulated mRNAs predominated at early times of starvation, especially at 4 h, whereas downregulated mRNAs attained near parity at 12 h and exceeded upregulated mRNAs at 24 h (Fig. 2E). The same trends were evident when we imposed a more stringent ±4-fold change to stratify the differentially expressed coding genes; in this case, there was a more pronounced lag time to close the numerical gap between upregulated and downregulated mRNAs (Fig. 2E, left panel).

In the ensuing sections, we address how inositol starvation affected the expression of genes involved in specific biological processes, as surmised from their known or imputed functions and their annotations in the fission yeast database PomBase. We focus on genes that were dysregulated at two or more time points in the experiment. The RNA-seq results are compiled in Tables S1 and S2. We also interrogate the physiological impact of inositol starvation on selected biological processes.

### Dysregulation of genes involved in lipid metabolism

**Fig 3:**
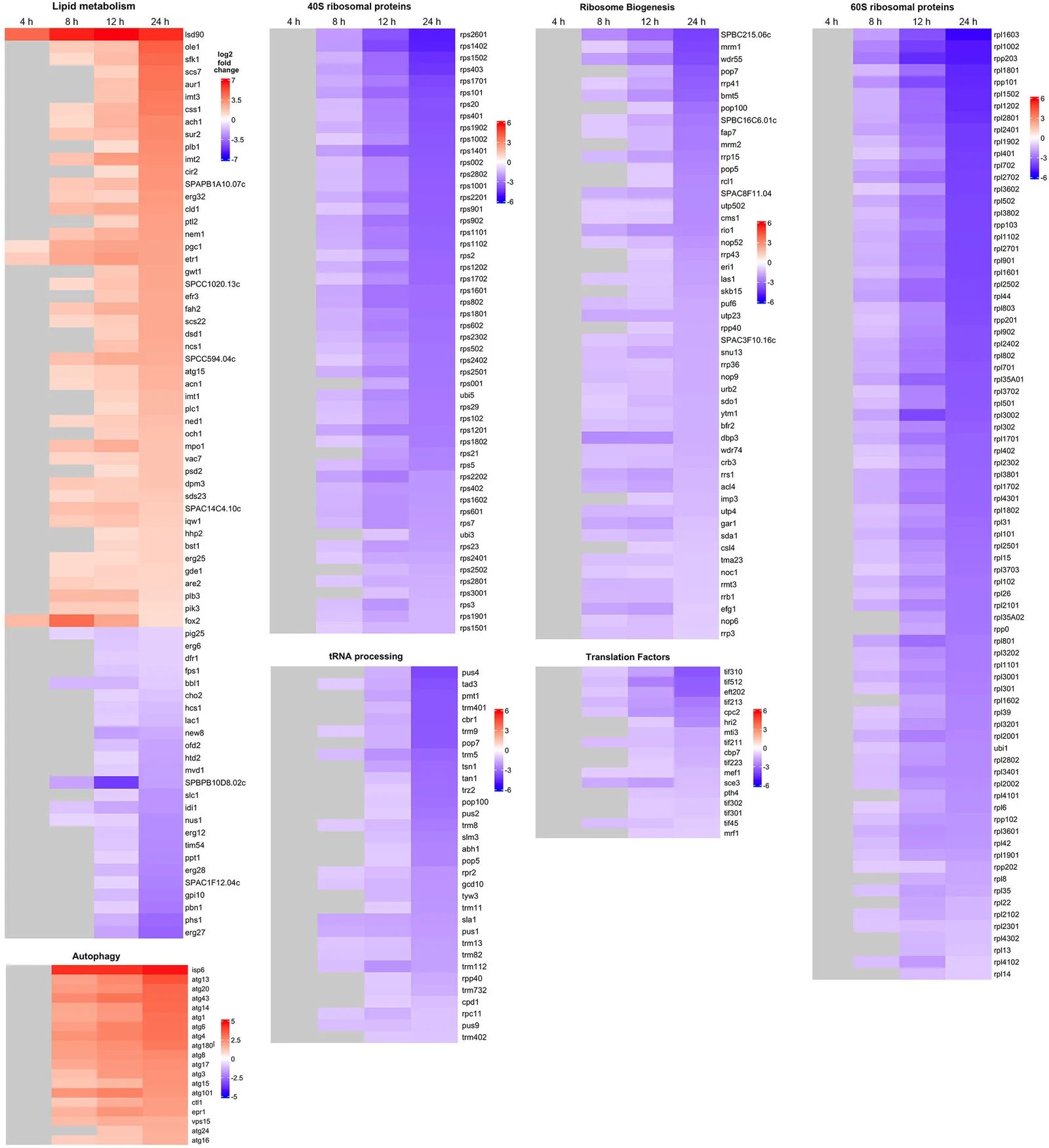
Inositol starvation-induced changes in mRNA expression affecting the indicated functional classes of genes. Each row represents a gene (specified at right), and each column represents the duration of inositol starvation. The log2 fold changes of individual mRNAs within the indicated functional classes of genes are colored according to the scales shown on the right. Red and blue signify upregulation and downregulation, respectively. Gray shading denotes less than a twofold change in transcript levels at the times specified.

Inositol starvation elicited upregulation of 48 genes and downregulation of 25 genes with known or imputed roles in lipid metabolism (Fig. 3; Table S2). Among the most highly upregulated transcripts were those encoding enzymes Ole1 (acyl-CoA desaturase; up 29-fold at 24 h), Scs7 (sphingosine hydroxylase; up 18-fold), Aur1 (inositol phosphorylceramide synthase; up 18-fold), Css1 (inositol phosphosphingolipid phospholipase C; up 15-fold), Ach1 (acetyl-CoA hydrolase; up 11-fold), Sur2 (sphingosine hydroxylase; up 11-fold), Plb1 (phospholipase B; up 11-fold); Erg32 (C-5 sterol desaturase; up 8-fold), Cld1 (cardiolipin phospholipase; up 8-fold), and Ptl2 (triacylglycerol lipase; up 7-fold). The most highly downregulated mRNAs included those encoding enzymes Erg27 (3-keto sterol reductase; down 14-fold at 24 h), Phs1 (3-hydroxyacyl-CoA dehydratase; down 13-fold), Pbn1 (glycosylphosphatidylinositol-mannosyltransferase subunit; down 10-fold), Ppt1 (para-hydroxybenzoate polyprenyltransferase; down 7-fold), Erg12 (mevalonate kinase; down 7-fold), and Nus1 (decaprenylcistransferase; down 7-fold). Transcripts encoding other lipid-metabolizing enzymes were dysregulated by 2- to 6-fold. The upregulated mRNAs in this category include several enzymes involved in inositol metabolism: Plc1 (phosphoinositide phospholipase C), Pik3 (phosphatidylinositol 3-kinase), Gde1 (glycerophosphodiester phosphodiesterase), Asp1 (inositol pyrophosphate kinase/pyrophosphatase) (Fig. 1), and Bst1 (GPI inositol deacylase). In addition, the transcript encoding the Itr2 inositol transporter is increased by fourfold in response to inositol starvation (Fig. 1). The mRNA encoding Arg82, the inositol polyphosphate kinase that converts IP_3_ to IP_5_, was downregulated by 16-fold (Fig. 1).

### Inositol starvation increases triacylglycerols and decreases phosphatidylinositols

**Fig 4:**
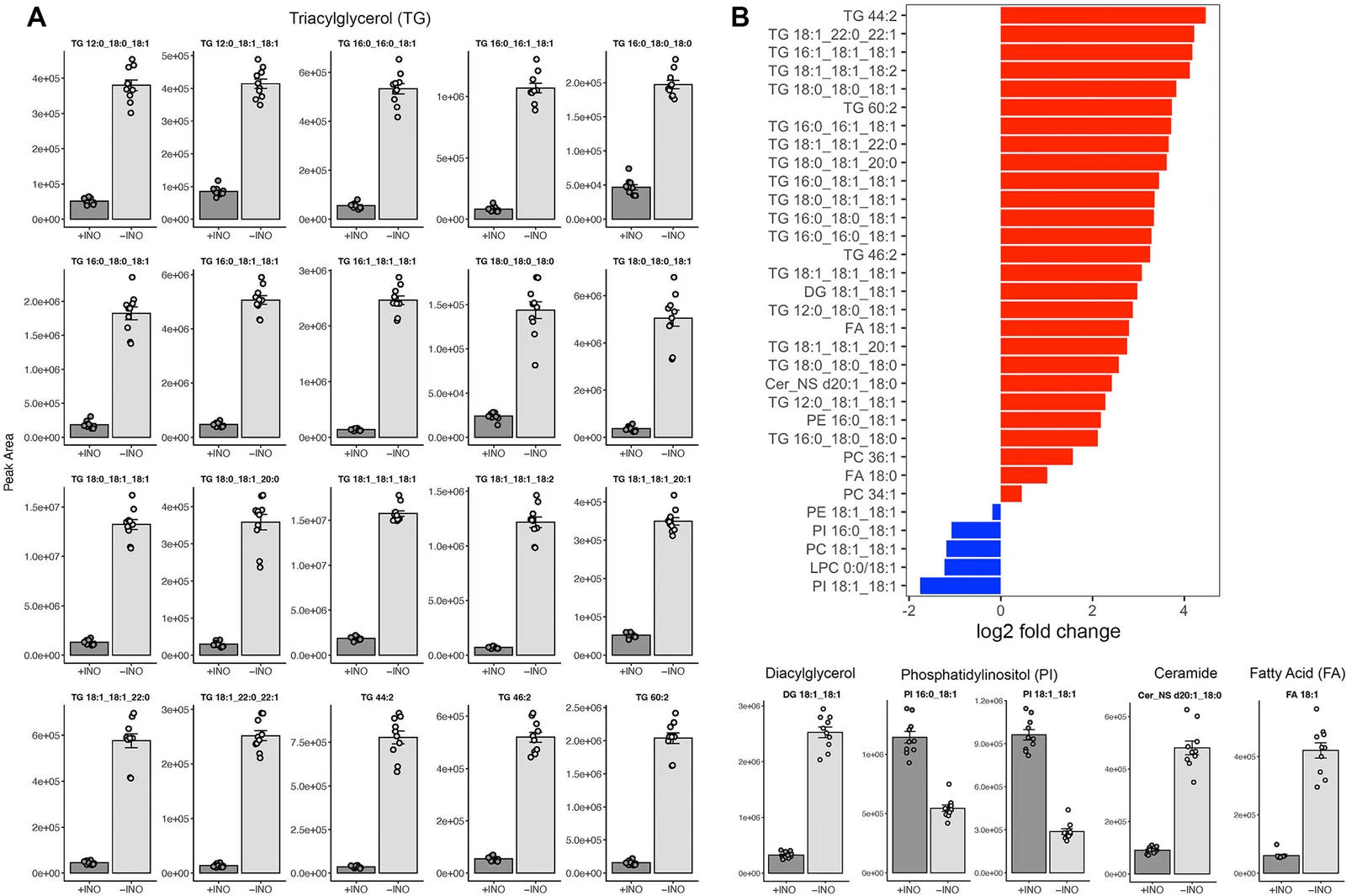
Inositol starvation increases triacylglycerols and decreases phosphatidylinositols. (**A**) The bar graphs show the levels (peak areas) of the indicated lipids in cells grown in inositol-replete medium (+INO) and cells maintained in inositol-free (–INO) medium for 24 h, as indicated on the *x*-axis. Each bar height is the mean of the individual peak areas (circle symbols; five biological replicates, each with one technical replicate) ± SD. (**B**) Log2 fold changes in lipid levels (cutoff of ±1 and *P* value of ≤0.05) are plotted in bar graph format.

We performed a lipidomics analysis of fission yeast cells from a mid-log culture in ePMGT(+INO) medium and from cells harvested 24 h after transfer to ePMGT(–INO) medium, entailing five biological replicates (independent cultures) and one technical replicate per biological replicate. We thereby identified by LC-MS 32 lipid species, comprising triacylglycerols (TG; *n* = 20), phosphatidylcholines (PC; *n* = 3), phosphatidylinositols (PI; *n* = 2), phosphatidylethanolamines (*n* = 2), fatty acids (FA; *n* = 2), lysophosphatidylcholine (LPC; *n* = 1), diacylglycerol (DG; *n* = 1), and ceramide (Cer; *n* = 1). Comparing the peak areas for inositol-replete and inositol-starved cells revealed that the levels of all 20 TGs, as well as the DG, FA, PE 16:0_18:1, and Cer species, were increased by 2- to 20-fold during inositol starvation (Fig. 4A and B). By contrast, the levels of PIs, LPC, and PC 18:1_18:1 decreased upon inositol starvation (Fig. 4A and B). Although not a complete survey of the fission yeast lipidome (14–16), our results highlight the theme that inositol starvation elicits a generalized accumulation of triacylglycerols and a modest depletion of phosphatidylinositol. A 1986 study of bulk phospholipid composition in fission yeast after 12 h of inositol starvation revealed a 15-fold decline in phosphatidylinositol and a threefold increase in phosphatidylserine, with no significant changes in phosphatidylcholine or phosphatidylethanolamine (5). It is conceivable that the increase in TGs reflects, at least in part, the fourfold increase in *ned1* mRNA encoding the enzyme phosphatidate phosphatase (Fig. 3). Ned1 conversion of phosphatidic acid to diacylglycerol is a crucial step in the synthesis of triacylglycerols (14). Note that DG 18:1_18:1 and TG 18:1_18:1 _18:1 increased to similar extents after inositol starvation (Fig. 4B).

### Inositol starvation depletes glycerophosphoinositol and affects lipid-adjacent metabolites

**Fig 5:**
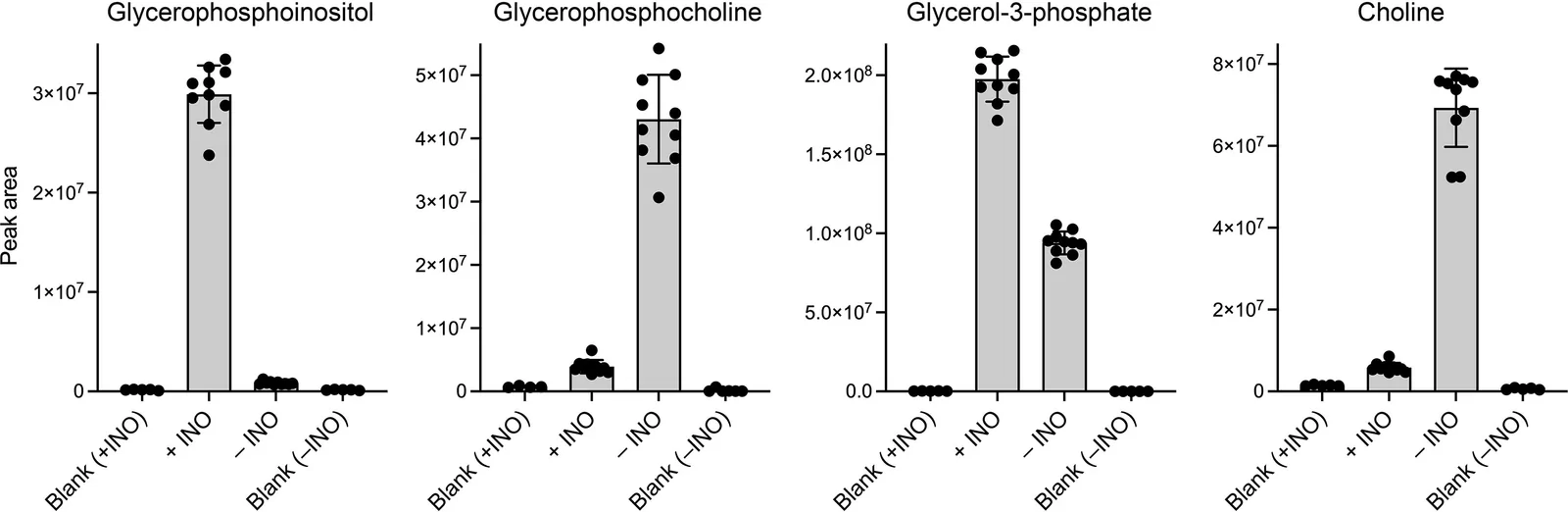
Inositol starvation depletes GPI and affects lipid-adjacent metabolites. The bar graphs show the levels (peak areas) of the indicated metabolites in cells grown in inositol-replete medium (+INO) and cells maintained in inositol-free (–INO) medium for 24 h, as indicated on the *x*-axis. Medium-only background levels are also shown. Each bar height is the mean of the individual replicate peak areas (circle symbols; five biological replicates, each with one technical replicate) ± SD.

Concomitant with the lipid analysis, we subjected the extracts of inositol-replete and inositol-starved cells to LC-MS profiling of polar metabolites, particularly key lipid-adjacent metabolites, such as GPI, glycerophosphocholine (GPC), glycerol-3-phosphate (G3P), and choline. GPI is generated by deacylation of phosphoinositide lipids (Fig. 1). GPC is produced by deacylation of phosphatidylcholine. GPC is hydrolyzed to choline and G3P by the enzyme Gde1, which also hydrolyzes GPI to inositol and G3P (Fig. 1). The mRNA encoding Gde1 increases by twofold during inositol starvation. We found that the cellular pool of GPI was depleted almost completely after 24 h of inositol starvation, i.e., reduced to 2% of the pre-starvation level (Fig. 5). By contrast, the level of GPC was increased by 13-fold after inositol starvation, and the level of choline was increased by 16-fold (Fig. 5). It would appear that inositol deficiency suppressed the catabolism of PI to GPI while increasing catabolism of PC to GPC and thence to choline. The level of G3P decreased by half after 24 h of inositol starvation (Fig. 5).

### Inositol starvation triggers depletion of 1,5-IP_8_

Inositol pyrophosphates 5-IP_7_, 1-IP_7_, and 1,5-IP_8_ are the most highly phosphorylated inositol metabolites; they function as signaling molecules that influence cellular phosphate homeostasis (17). In fission yeast, 1,5-IP_8_ and 1-IP_7_ impact gene expression by acting as agonists of RNA 3′-processing and transcription termination, especially affecting the lncRNAs that regulate expression of phosphate acquisition genes *pho1*, *pho84*, and *tgp1* (18, 19). 1,5-IP_8_ is also an allosteric activator of vacuolar inorganic polyphosphate synthesis by the fission yeast VTC complex (20, 21). 1,5-IP_8_ is synthesized by position-specific kinases Kcs1 and Asp1 that convert IP_6_ to 5-IP_7_ and 5-IP_7_ to 1,5-IP_8_, respectively (Fig. 1). Asp1 is a bifunctional kinase/pyrophosphatase that also hydrolyzes IP_8_ back to 5-IP_7_ (22). Two other inositol pyrophosphatase enzymes, Aps1 and Siw14, join Asp1 as agents of inositol pyrophosphate catabolism (23–25). Recent studies showed that the cellular pool of inositol pyrophosphates is rapidly depleted in response to acute phosphate starvation (26, 27). To wit, acute phosphate starvation resulted in progressive depletion of 1,5-IP_8_, 5-IP_7_, and 1-IP_7_ at 1, 2, 4, and 6 h, by which time they were reduced to 4%, 8%, and 7% of the pre-starvation levels, respectively (27). The effects of inositol deprivation on inositol pyrophosphates are currently unknown.

**Fig 6:**
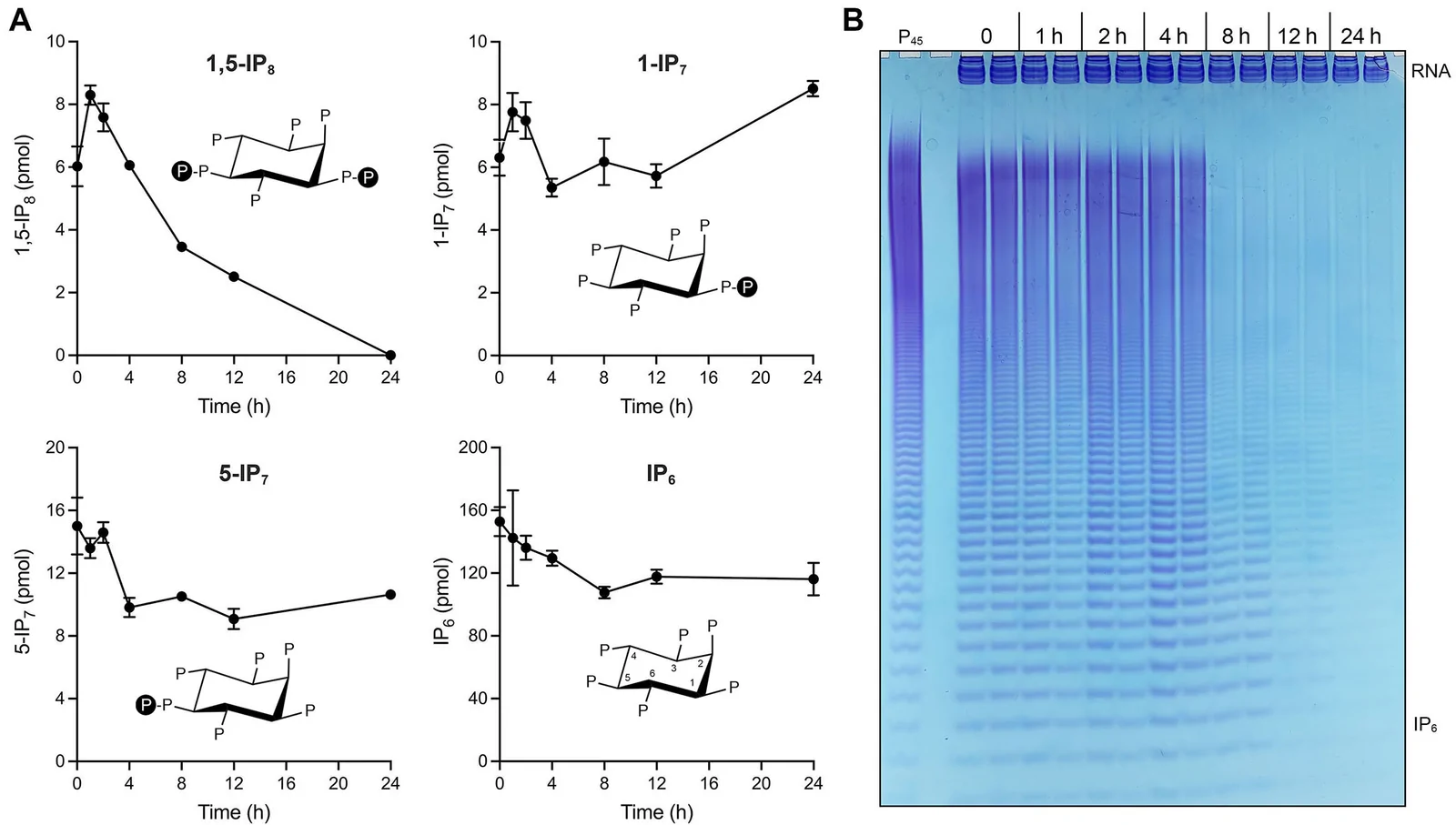
Inositol starvation triggers depletion of 1,5-IP_8_ and inorganic polyphosphate. (**A**) The amounts (pmol) of 1,5-IP_8_, 5-IP_7_, 1-IP_7_, and IP_6_ in equal volumes of extracts of 10 *A*_600_ units of cells are plotted as a function of time of inositol starvation. The data shown are the average of three biological replicates ± SEM. The chemical structures of 1,5-IP_8_, 5-IP_7_, 1-IP_7_, and IP_6_ are depicted in the respective graphs. (**B**) Total polyphosphate prepared from inositol-replete (time 0) and inositol-starved cells was analyzed by PAGE and visualized by staining with toluidine blue. The times of inositol starvation are specified above the lanes. Two biological replicates are shown for each time point. The position of IP_6_ analyzed in parallel is indicated on the right. The toluidine blue-stained material just below the sample wells is RNA.

To fill this knowledge gap, we deployed capillary electrophoresis electrospray ionization mass spectrometry (CE-ESI-MS) to profile the pool of 1,5-IP_8_, 5-IP_7_, and 1-IP_7_ in fission yeast cells prior to (time 0) and at 1, 2, 4, 8, 12, and 24 h after inositol starvation. Whole-cell perchloric acid extracts from 10 *A*_600_ units of cells were adsorbed to titanium dioxide beads to enrich for inositol polyphosphates (28). CE-ESI-MS was performed as described (25, 29) using ^13^C-labeled internal standards (30). The amounts (in pmol) of 1,5-IP_8_, 5-IP_7_, and 1-IP_7_ in each sample were determined and are plotted in Fig. 6A as the average of three biological replicates (independent cultures). We found that inositol starvation resulted in a time-dependent depletion of 1,5-IP_8_ between 4 and 24 h, whereby the IP_8_ levels after 4, 8, 12, and 24 h were 100%, 57%, 42%, and <1% of the pre-starvation level, respectively. By contrast, 5-IP_7_, 1-IP_7_, and IP_6_ were relatively unaffected by inositol deprivation; their levels after 24 h were 71%, 135%, and 76% of the pre-starvation levels, respectively.

### Depletion of inorganic polyphosphate stores during inositol starvation

Inorganic polyphosphate (polyP) resident in the yeast vacuole serves as a phosphate reservoir in times of need. PolyP is produced by a membrane-associated VTC complex—composed of Vtc4, Vtc2, and Vtc1 subunits—that synthesizes polyP and simultaneously imports the polyP into the yeast vacuole (31–34). Vtc4 is the catalytic subunit of the polyP polymerase; it consists of a cytoplasm-facing N-terminal SPX domain, a central cytoplasmic polymerase domain of the triphosphate tunnel metalloenzyme family, and a C-terminal transmembrane domain. The SPX domain binds 1,5-IP_8_, which stimulates polyP synthesis by fission yeast VTC (20, 21).

Here, we monitored the fate of the polyP pool during inositol starvation via polyacrylamide gel electrophoresis and staining with toluidine blue (21). PolyP is resolved into a ladder of linear chains, with each step differing by one phosphate unit. Whereas the polyP profile was unchanged after 4 h of inositol starvation, the chain length of the linear polyP species was shortened after 8 h; polyP content declined further at 12 and 24 h (Fig. 6B). Thus, the vacuolar polyP reservoir is gradually depleted, in sync with depletion of 1,5-IP_8_ that drives polyP synthesis by VTC. It is worth noting that polyP is exhausted more quickly during phosphate starvation than inositol starvation, i.e., polyP is shortened after 1 h of phosphate starvation and is undetectable after 2–3 h (9), in keeping with the more rapid decline in 1,5-IP_8_ during phosphate starvation versus inositol starvation (27).

### Upregulation of genes involved in autophagy

Autophagy is a response to cellular stress and nutrient limitation that entails enclosure of cytoplasmic components within autophagosomes that fuse with lysosomes/vacuoles, wherein degradation of pre-existing macromolecules helps salvage and restore nutrients (35). Expression of fission yeast autophagy genes (36) is upregulated in response to diverse cellular stressors, including phosphate starvation (9, 37–39). We find here that 19 of the fission yeast genes in the autophagy pathway were upregulated at the transcriptional level in response to inositol starvation, 18 of which were induced at 8 h and all of which were upregulated by 2- to 26-fold at 12 and 24 h (Fig. 3; Table S2).

### Global downregulation of the translation machinery

The mRNAs encoding 52 proteins of the 40S ribosome and 80 proteins of the 60S ribosome were downregulated at 8 to 24 h post-starvation, by 3- to 44-fold (Fig. 3; Table S2). Inositol starvation reduced (by 2-fold to 13-fold, at 8 or 12 to 24 h) the expression of 13 genes encoding cytoplasmic translation factors and 4 mitochondrial translation factors (Fig. 3; Table S2).

Inositol starvation dampened (by 2- to 16-fold) the expression of six genes encoding protein components of the fission yeast RNA polymerase I (Pol1) transcription machinery responsible for synthesis of pre-rRNA (Table S2). These include Pol1-specific subunits Rpa43 and Ker1; Rpc19, a Pol1 subunit shared with Pol3; Rpb8 and Rpb10, subunits shared with Pol3 and Pol2; and Pol1-specific initiation factor subunit Rrn10. (By contrast, the mRNA for Pol1 initiation factor Rrn3 was upregulated by sevenfold.) pre-rRNA undergoes a series of processing steps to generate the mature 18S, 28S, and 5.8S rRNAs, which is coupled to the assembly of 40S and 60S ribosomes in the nucleolus. The mRNAs for 50 proteins involved in ribosome assembly and maturation were downregulated by 2- to 17-fold during inositol starvation (Fig. 3; Table S2).

### Dysregulation of genes involved in tRNA biogenesis and function

RNA polymerase III (Pol3) is responsible for the synthesis of tRNAs and 5S rRNA. Inositol starvation downregulated by 3- to 16-fold the expression of genes encoding five of the subunits of fission yeast Pol3 (Pol3-specific proteins Rpc37 and Rpc11, shared subunits Rpc10, Rpc19, and Rpb8) and TFIIIC subunit Sfc6 (Table S2). By contrast, the genes for the TFIIIC subunit Sfc3, TFIIIB subunits Bdp1 and Brf1, and the Pol3 transcription repressor Maf1 were upregulated by 2- to 6-fold (Table S2). Upregulation of Sfc3, Brf1, Bdp1, and Maf1 is also observed during phosphate starvation of fission yeast (9, 27).

In addition to affecting the machinery that transcribes pre-tRNAs, inositol starvation resulted in downregulation (by 2- to 14-fold) of 32 genes encoding proteins that process and modify tRNAs (Fig. 3; Table S2). Inositol starvation also elicited a 3- to 14-fold decrement in mRNAs encoding 11 aminoacyl tRNA synthases (6 cytoplasmic and 5 mitochondrial) (Table S2).

### Inositol starvation impacts polysomes differently than phosphate starvation

**Fig 7:**
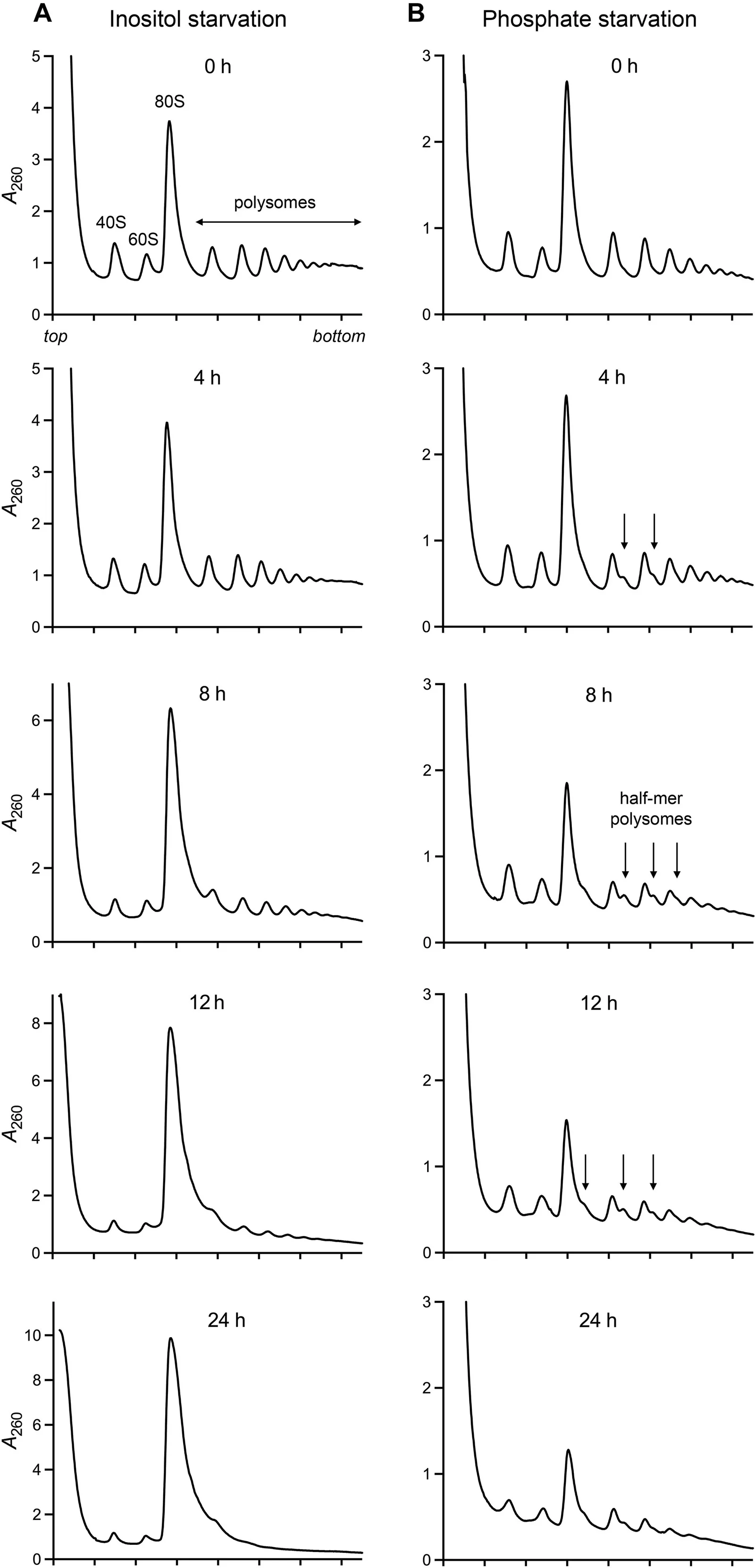
Inositol starvation impacts polysomes differently than phosphate starvation. Sucrose gradient analyses of ribosomes and polysomes are shown for fission yeast cells harvested prior to (time 0) and after 4, 8, 12, and 24 h of inositol starvation (**A**) or phosphate starvation (**B**). Half-mer polysomes in phosphate-starved cells are denoted by vertical arrows.

To interrogate the functional impact of the downregulation of translation-related genes, we performed sucrose gradient sedimentation analyses of ribosomes and polysomes from lysates of cells harvested prior to (time 0) and at 4, 8, 12, and 24 h after the onset of inositol starvation. Free 40S and 60S subunits were well resolved from 80S monosomes and polysomes containing between 2 and 8 or more ribosomes in the 0 h samples (Fig. 7A). Whereas there was no discernible change in the profile after 4 h of inositol starvation, cells starved for 8, 12, and 24 h evinced a progressive dissolution of polysomes and a compensatory increase in 80S monosomes, with little change in the abundance of free 40S and 60S subunits (Fig. 7A).

For comparison, we performed a parallel analysis of ribosomes and polysomes from lysates of cells harvested prior to (time 0) and at 4, 8, 12, and 24 h after the onset of phosphate starvation (Fig. 7B), which revealed a different fate for the translation machinery. At 4 h of phosphate starvation, we noted the appearance of “half-mer” polysomes, seen as shoulder peaks on the heavy side of the monosome, disome, and trisome peaks (Fig. 7B). Half-mer polysomes comprise a single stalled 40S subunit, followed by one or more complete 80S ribosomes engaged on an mRNA. They are taken to be a sign of decreased ribosome fitness (40–46). The half-mer polysomes persisted at 8, 12, and 24 h of phosphate starvation, even as the overall abundance of polysomes and monosomes declined at 12 and 24 h. These results highlight that inositol starvation and phosphate starvation exert distinctive effects on the translation machinery.

### RNA polymerase II (Pol2) transcription factors

**Fig 8:**
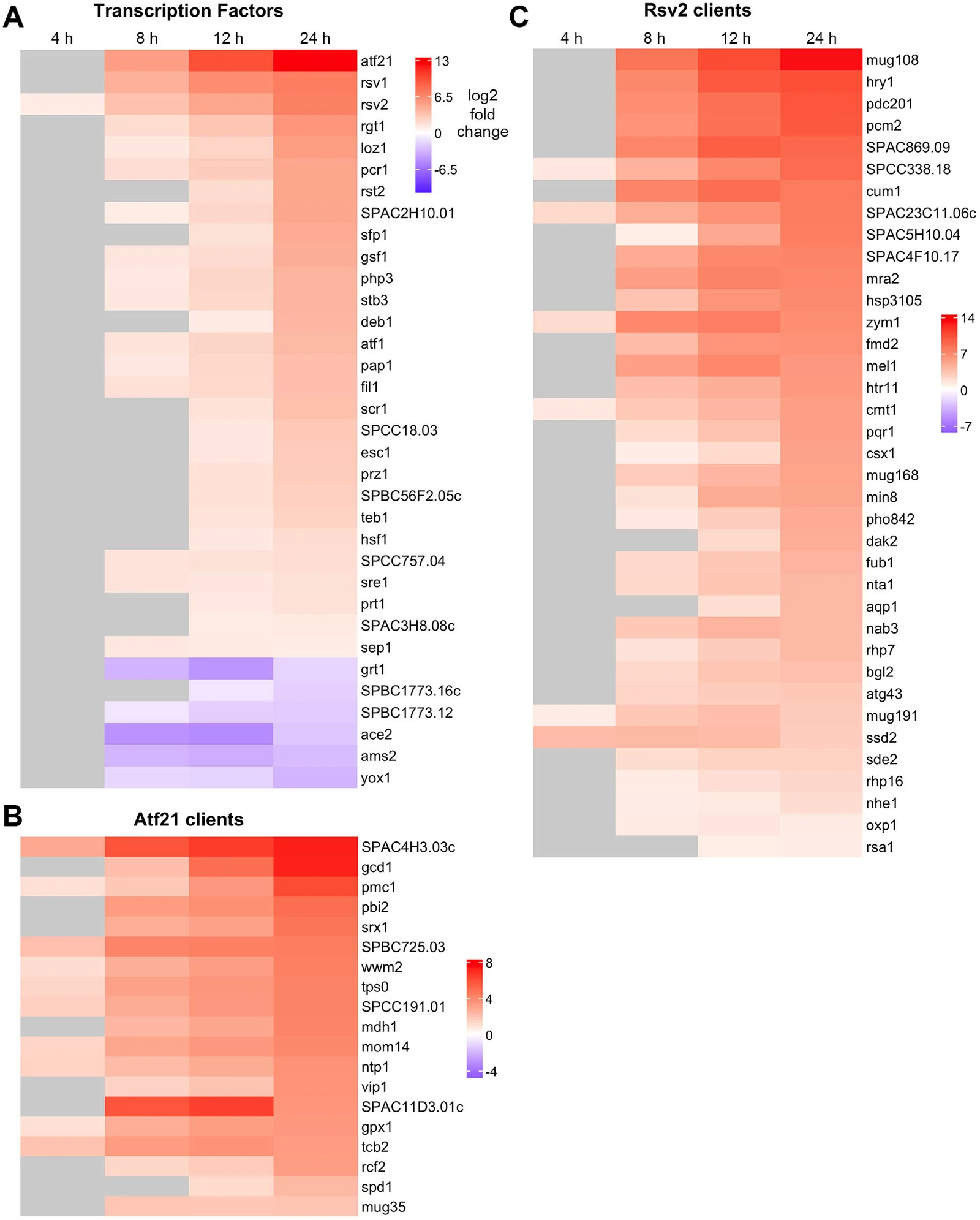
Expression of mRNAs encoding RNA polymerase II transcription factors and client genes. (**A**) Heat map of log2 fold changes in mRNAs encoding transcription factors. Each row represents a transcription factor gene (specified at right). Each column represents the duration of inositol starvation. (**B**) Expression of Atf21-responsive genes (48) during inositol starvation. (**C**) Expression of Rsv2-responsive genes (48) during inositol starvation. The log2 fold changes of individual mRNAs within the indicated functional classes of genes are colored according to the scales shown on the right. Red and blue signify upregulation and downregulation, respectively. Gray shading denotes less than a twofold change in transcript levels at the times specified.

Six DNA-binding Pol2 transcription factor genes were downregulated during inositol starvation, with the most affected being the gene for the C2H2 zinc finger transcription activator Ace2 (down by 29- to 37-fold at 8 to 12 h) (Fig. 8A). By contrast, *ace2* mRNA transiently increases during phosphate starvation: by 7-fold and 4-fold after 4 and 12 h, respectively, before returning to basal level at 24 h (6). Ace2 positively controls the expression of seven genes (*adg1*, *adg2*, *adg3*, *cfh4*, *agn1*, *eng1*, *mid2*) involved in septation and cell separation (47). We found that the Ace2 client genes were themselves downregulated during inositol starvation: *eng1* by 98-fold; *adg1* by 23-fold; *agn1* by 11-fold; *adg2* by 9-fold; *mid2* by 6-fold; *adg3* by 4-fold; *cfh4* by 3-fold.

Twenty-eight different Pol2 DNA-binding transcription factors were upregulated during inositol starvation (Fig. 8A). The top “hits” in this category based on increase at 24 h were *atf21* (up 6,800-fold), *rsv1* (150-fold), *rsv2* (130-fold), *rgt1* (60-fold), and *loz1* (44-fold). Atf21 is a bZIP (basic-leucine zipper) family transcription factor that is upregulated during a variety of cellular stresses: amino acid starvation, cadmium exposure, DNA damage, heat, and osmotic stress (37, 38). Atf21 overexpression was evident after 8 h of inositol starvation (up 43-fold) and continued to increase at 12 and 24 h (Fig. 8A). By contrast, Atf21 expression was upregulated only transiently during acute phosphate starvation, by eightfold at 4 h, and returned to baseline after ≥12 h (9). Forced overexpression of Atf21 in vegetative cells elicited upregulation of 26 protein-coding genes, as gauged by microarray analysis (48). Here, we found by RNA-seq that 18 of the Atf21 client genes were upregulated during inositol starvation (Fig. 8B), most prominently *SPAC4H3.03c* (glucan alpha-1,4-glucosidase, up 155-fold), *gcd1* (glucose dehydrogenase, 150-fold), *SPAC11D3.01c* (Con-6 family protein, 84-fold), *pmc1* (P-type ATPase, 68-fold), *pbi2* (vacuolar protease inhibitor, 32-fold), and *srx1* (sulfiredoxin, 27-fold) (Table S1).

Rsv1 is a C2H2 zinc finger protein described as a transcription repressor based on the findings that 24 genes were expressed at higher levels in vegetative *rsv1*∆ cells versus *rsv1*^+^ cells (48). Thus, we might expect that the increase in *rsv1* mRNA during inositol starvation would lead to decreased expression of the putative Rsv1 client genes. Indeed, we found that Rsv1 clients *efn1* and *efn2* were repressed by 22-fold and 24-fold, respectively, at 24 h of inositol starvation. Efn1 and Efn2 are extracellular 5′-nucleotidase enzymes that are massively transcriptionally upregulated and secreted into the medium during phosphate starvation, where they comprise the most abundant proteins in the “secretome” and act to mobilize inorganic phosphate from extracellular NMPs that can then be imported into phosphate-starved cells (9, 49). However, other genes flagged as potential Rsv1 targets (48) were strongly upregulated during inositol starvation—including hexose transporter genes *ght1* (by 10-fold), *ght3* (48-fold), and *ght4* (43-fold), and genes *wwm3* (WW domain protein, up 3,000-fold) and *dak2* (dihydroxyacetone kinase, 29-fold) (Table S1)—which suggests either that they are not direct targets of repression by Rsv1 or that other activating transcription factors prevail at these genes during inositol starvation.

Rsv2 is a C2H2 zinc finger transcription factor induced during cellular stress (9, 37, 38). Rsv2 is deemed a transcription activator in light of the finding that the expression of 39 protein-coding genes was reduced in *rsv2*∆ cells undergoing sexual differentiation versus *rsv2*^+^ cells (48). Here, we found that 37 of these Rsv2 client genes were upregulated during inositol starvation (Fig. 8C). The greatest increases were seen for *mug108* (unknown function, up by 9,400-fold), *hry1* (iron-sulfur cluster repair protein, 1,500-fold), *pdc201* (pyruvate decarboxylase, 1,100-fold), *pcm2* (protein-L-isoaspartate O-methyltransferase, 1,000-fold), *SPAC869.09* (Con-6 family protein, 800-fold), *cum1* (Con-6 family protein, 430-fold), *SPCC338.18* (pombe specific protein, 460-fold), *zym1* (metallothionein, 220-fold), *SPAC5H10.04* (NADPH dehydrogenase, 220-fold), *mra2* (ATP synthase assembly protein, 170-fold), *SPAC4F10.17* (conserved fungal protein, 170-fold), and *mel1* (alpha-galactosidase, 140-fold).

Loz1 is a C2H2 zinc finger transcriptional repressor that inhibits the expression of 27 genes when zinc is in excess (50). We find here that the increased *loz1* expression during inositol starvation is correlated with decreased expression of five of the Loz1 client genes, including two of known function: *adh4* (alcohol dehydrogenase, down by 18-fold) and *zrt1* (plasma membrane zinc transporter, 14-fold). The other three Loz1-responsive genes that are repressed during inositol starvation*—SPBPB2B2.15*, *SPBC1348.06c*, and *SPAC977.05c*—encode three nearly identical paralogous proteins (203 or 204 amino acid polypeptides) annotated in PomBase as members of a “velum formation protein 1-like conserved fungal family.” These three genes are the most highly repressed coding transcripts identified in our inositol starvation RNA-seq analysis (down by 300- to 500-fold; Table S1), and they are the most highly induced genes in response to zinc limitation (51). *SPBC1348.06c* is annotated as an essential gene, whereas the other two are inessential. Their function is not known. It is conceivable that the decline in inorganic polyphosphate that occurs during inositol starvation leads to an increase in free zinc—vacuolar polyphosphate functions as a divalent metal chelator—that in turn triggers the increase in *loz1* expression. However, this simple scenario is inconsistent with the finding that *loz1* expression is not increased during phosphate starvation, despite the complete depletion of inorganic polyphosphate in phosphate-starved cells (9).

### Changes in *PHO* gene expression during inositol starvation

*S. pombe* adapts to acute phosphate starvation by upregulating the expression of a battery of genes encoding proteins that mobilize and import phosphate from the extracellular milieu, including (i) cell-surface acid phosphatase Pho1 that releases phosphate from phosphomonoesters; (ii) secreted extracellular 5′-nucleotidase enzymes Efn1 and Efn2 that release phosphate from NMPs; and (iii) plasma membrane phosphate transporters Pho84, Pho841, and Pho842. Induction of *pho1*, *efn1*, *pho84*, and *pho842* expression during phosphate starvation is attenuated in cells lacking transcription factor Pho7 (8, 10). Here, we find that inositol starvation led to decreased expression of *pho1* (by 26-fold), *efn1* (22-fold), and *pho84* (32-fold). By contrast, inositol starvation increased expression of *pho841* (42-fold) and *pho842* (32-fold) (Table S1) (note that *pho7* mRNA levels did not change during either phosphate or inositol starvation). We surmise that phosphate-responsive genes are subject to distinct transcriptional controls during phosphate and inositol scarcity.

## DISCUSSION

We characterized the cellular adaptations of fission yeast to inositol starvation, entailing integration of genome-wide analysis of changes in mRNA levels and tracking the status of inositol-adjacent metabolites. Our results highlight distinctive features of the inositol starvation response, as well as commonalities *vis-à-vis* other stresses. It was of particular interest to us to compare the effects of inositol and phosphate deprivation, given that most downstream metabolites of inositol are phosphorylated or polyphosphorylated.

Cells starved for either phosphate or inositol cease growth after two or three rounds of division, albeit with different outcomes with respect to the cell cycle. Phosphate starvation elicits a durable state of G0 quiescence populated by a near-equal mix of cells with 1C and 2C DNA content (9). Cells starved for inositol pile up with 4C DNA content, and we see no cells with 1C DNA content, which suggests a G2 arrest. Phosphate-starved cells display a relatively resilient chronological lifespan, with no loss of viability for the first 2 days, followed by a steady decline in survival over 28 days (9). Inositol-starved cells maintain full viability for 8 to 12 h before suffering 80% attrition by 24 h.

Transcriptome changes evolved over time of inositol starvation, both with respect to the number of mRNAs dysregulated and, in most cases, the magnitude of the increase or decrease in mRNA abundance. Upregulated genes outnumbered downregulated genes at early times. Several coherent whole-pathway alterations in gene expression were observed during inositol starvation that echo those reported for phosphate starvation and a variety of other cellular stresses (9, 37, 38, 52). The roster of stress-responsive genes, initially designated core environmental stress response genes (52), has grown as the sensitivity of available tools for transcriptomics has progressed from microarrays to RNA-seq. These include (i) upregulation of genes in the autophagy pathway; and (ii) downregulation of genes for the biogenesis and function of ribosomes and tRNAs.

The impact of sustained downregulation of genes related to protein synthesis on the translation apparatus was manifest by conversion of polysomes to 80S monosomes during the interval from 8 to 24 h of inositol starvation, which coincided with the decrement in expression of translation-related genes. Our results resonate with recent reports that 80S monosome accumulation occurs during adaptation to other cellular stresses, e.g., glucose depletion and heat shock (53–55). The present sucrose gradient analysis does not discern if the 80S monosomes that accumulate during inositol starvation are actively engaged in polypeptide elongation or are translationally silent and devoid of tRNA or mRNA. It will be of interest in future studies to interrogate the structures of the 80S monosomes that accumulate in inositol-starved cells and the mRNAs with which they are associated. It is noteworthy that phosphate starvation exerts a quite different impact on the translation apparatus, whereby polysomes persist but display an apparent defect in the union of free 60S subunits with 40S subunits engaged on mRNA, as reflected by the appearance of half-mer polysomes. A potential source of this difference is that the global downregulation of ribosomal protein genes during the initial 12 h of phosphate starvation is transient, reaching a nadir at 8 h and then recovering by 24 h (9, 10).

Given the key roles of inositol metabolites in cell signaling and lipid membrane structure and function, it is not surprising that inositol starvation elicited widespread changes in the expression of genes involved in lipid metabolism and inositol polyphosphate dynamics. We were especially interested in the effects of inositol starvation on inositol pyrophosphates, which figure prominently in fission yeast phosphate homeostasis and are depleted during phosphate starvation. We find that inositol starvation results in selective depletion of 1,5-IP_8_, with little impact on the levels of 1-IP_7_ and 5-IP_7_. The kinetics of 1,5-IP_8_ depletion track with the depletion of the vacuolar reserve of polyP. This is a significant finding, insofar as previous genetic studies had established that the Asp1 kinase, which is responsible for synthesis of 1,5-IP_8_ from 5-IP_7_ and of 1-IP_7_ from IP_6_, is a key driver of vacuolar polyP synthesis by fission yeast Vtc4, which contains an inositol pyrophosphate-sensing SPX domain (20, 21). The fact that both inositol-1-pyrophosphate species decline in parallel during phosphate starvation in tandem with depletion of polyP (27) did not resolve the question of whether 1,5-IP_8_ or 1-IP_7_ is the principal activator of polyP synthesis. The present results weigh in favor of 1,5-IP_8_ as the relevant determinant in wild-type fission yeast cells.

An initial lipidomics analysis reveals that 24 h of inositol starvation causes an across-the-board elevation of all 20 triacylglycerol species we were able to assign, as well as a decline in two phosphatidylinositols. We presume that changes in the lipidome are driven, at least in part, by the changes in mRNAs encoding enzymes of lipid synthesis and catabolism. We speculate that elevated Ned1 expression might contribute to the accumulation of triacylglycerols. However, given the many moving parts that are impacted at the level of gene expression, it is difficult to pinpoint a particular enzymatic step as causal. (The same caveat pertains to assigning the changes in ribosome distribution to altered expression of specific ribosome components.)

Important clues to the origins of the transcriptome remodeling during inositol starvation emerged from the large-magnitude changes in mRNAs encoding certain RNA polymerase II transcription factors for which prior studies had identified genes under their presumed direct control, via studies entailing transcription factor overexpression or deletion. The most dramatic case in our present analysis involved Atf21 mRNA, which was nearly undetectable at baseline but increased by 6,800-fold during inositol starvation (read counts of 18 at time 0 versus 124,000 at 24 h). Our RNA-seq results highlighted corresponding increases in the expression of 18 of the putative Atf21 client genes, thereby underscoring the existence of a bona fide Atf21 regulon activated in response to a specific cellular stress. We also affirm the existence of an inositol starvation-responsive Rsv2 regulon that embraces 37 of the suggested Rsv2 client genes. It will be of interest in future studies to interrogate the impact of deletions of the most highly dysregulated transcription factor genes (e.g., *atf21*∆, *rsv2*∆, *rsv1*∆, *loz1*∆, *rgt1*∆, *pcr1*∆, *ace2*∆) on the physiology of the fission yeast inositol starvation response.

A major open question is whether there are regulon-level signaling pathways that trigger the observed dysregulation of lipid-related genes during inositol starvation and, if so, the identity of the sensors and transcription factors that bring about those changes. In the same vein, it remains to be determined which transcription factors might be responsible for the upregulation of autophagy genes and the downregulation of protein synthesis-related genes during the core environmental stress response. Chen et al. (52) suggested that bZIP transcription factor Atf1 might be a master regulator of stress-responsive genes in fission yeast. We find that *atf1* mRNA is upregulated by 3-fold, 5-fold, and 14-fold after 8, 12, and 24 h of inositol starvation (Fig. 8A). However, the genome-wide map of Atf1’s physical interaction with promoters reported by Skribbe et al. (56) did not detect Atf1 at the promoters of autophagy genes (or of protein-synthesis-related genes). It might be the case that promoter engagement of the factors driving increased expression of lipid and autophagy genes is contingent on either a protein modification or a second messenger generated by inositol starvation. With respect to downregulated gene expression during starvation, we know that virtually all of the fission yeast ribosomal protein genes, as well as several translation factor genes, contain a HomolD sequence element in their promoters (9, 57). However, the transcription factor that activates HomolD promoters *in vivo* has not been identified. It remains to be seen if the repression of ribosomal protein and transcription factor genes during cellular stress entails inactivation of the HomolD-binding transcription factor, versus production of a separate factor that represses the HomolD-driven genes.

## MATERIALS AND METHODS

### Inositol starvation

Fission yeast strains were grown at 30°C in ePMGT medium (9, 10), which contains 55 µM inositol, to an *A*_600_ of 0.5–0.7. The cells were harvested by centrifugation, washed with ePMGT medium without inositol, and resuspended at an *A*_600_ of 0.25 in ePMGT(–INO). The inositol-free cultures were incubated at 30°C and diluted with ePMGT(–INO), not to exceed an *A*_600_ of 0.8. At the times specified (i) a volume of culture containing 15 *A*_600_ units of cells was rapidly cooled by introducing it into ice and cells were harvested by centrifugation at 4°C; the cells were then transferred to 1.5 mL screw-cap tubes and then frozen on dry ice for subsequent RNA analyses; (ii) an aliquot of cells was fixed in 70% ethanol and DNA content was measured by flow cytometry according to the basic protocol as described (11); and (iii) cells were observed by light microscopy.

### RNA-seq analysis

Total RNA was extracted via the hot phenol method (58) from 15 *A*_600_ units of wild-type cells harvested prior to (time 0) and at 4, 8, 12, and 24 h after transfer to ePMGT(–INO) medium. Three biological replicates were sequenced for each time point. The integrity and quantity of total RNA were gauged with an Agilent 4200 TapeStation system. The NEB Ultra II Directional RNA Library Prep plus Poly A isolation module kit was used to prepare the libraries for paired-end sequencing using a NovaSeq 6000 system. FASTQ files bearing paired-end reads of 51 bases in length (total paired reads of 29.7 million to 40.5 million per biological replicate) were mapped to the *S. pombe* genome using HISAT2-2.1.0 with default parameters (59). Mapped reads comprised 98% of the total reads per replicate (Fig. S1). The resulting SAM files were converted to BAM files using Samtools (60). Count files for individual replicates were generated with HTSeq-0.10.0 (61) using exon annotations from Pombase (GFF annotations, genome version ASM294v2; source “ensembl”). RPKM analysis and pairwise correlations (Pearson coefficients of 0.980 to 0.991; Fig. S1) were performed as described previously (62). Differential gene expression and fold change analysis were performed using DESeq2 (63). The cutoff for further evaluation was set for genes with an adjusted *P* value (Benjamini-Hochberg corrected) of ≤0.05, which were either upregulated or downregulated by at least twofold at two or more starvation time points compared to the time 0 control. Genes were further filtered based on the following criteria: (i) genes that were ≥2-fold upregulated and had an average normalized read count at a given time point of ≥100; and (ii) genes that were ≥2-fold downregulated and the average normalized read count at time 0 was ≥100.

### Analysis of inositol pyrophosphates by capillary electrophoresis electrospray ionization mass spectrometry

Aliquots of fission yeast cells (10 *A*_600_ units, corresponding to 2 to 3 × 10^8^ cells) grown at 30°C were harvested prior to (time 0) and at sequential times after transfer from liquid ePMGT(+INO) to ePMGT(–INO) medium. The cells (three biological replicates for each strain and starvation time) were washed in ice-cold water and resuspended in 1 mL of 1 M perchloric acid. After snap-freezing in liquid nitrogen, the samples were thawed, mixed briefly by vortexing, and cell debris was removed by centrifugation at 16,200 × *g* for 5 min at 4°C. Acid-extracted inositol polyphosphates in the supernatants were then purified using titanium dioxide beads (25, 28). Inositol polyphosphate concentrations in the TiO_2_-enriched cell extracts were determined by CE-ESI-MS with an Agilent 7100 capillary electrophoresis (CE) system coupled to a triple quadrupole tandem mass spectrometry Agilent 6495c system (25, 29). The levels of 1,5-IP_8_, 1-IP_7_, and 5-IP_7_ are reported in the figures as total pmol in the 30 µL sample of TiO_2_-enriched cell extract (three biological replicates ± SEM).

### PAGE assay of intracellular polyphosphate content

Aliquots of fission yeast cells (5 *A*_600_ units, corresponding to ~1.2 × 10^8^ cells) grown at 30°C were harvested by centrifugation prior to (time 0) and at sequential times after transfer from liquid ePMGT(+INO) to ePMGT(–INO) medium. The cells were washed with cold water, recentrifuged, and stored at −80°C. The cell pellets were resuspended in 400 µL AE buffer (50 mM sodium acetate, pH 5.2, 10 mM EDTA) and added to 400 µL phenol (equilibrated in 10 mM Tris-HCl, pH 8.0) plus 40 µL of 10% (wt/vol) SDS. The samples were mixed vigorously, incubated at 65°C for 10 min, and then placed on ice. After centrifugation at 4°C (10 min at 17,000 × *g*), the aqueous phase (~400 µL) was collected and extracted once with phenol/chloroform and then with chloroform. Ethanol (1 mL) was added to precipitate polyP (and any nucleic acids) during incubation at −20°C overnight. The precipitates were washed with 70% ethanol, dried, and resuspended in 50 µL TE (10 mM Tris-HCl, pH 7.0, 1 mM EDTA; 10 µL per 1 *A*_600_ unit of cells). Aliquots (8 µL) were adjusted to 10% (vol/vol) glycerol and then analyzed by electrophoresis through a 30% polyacrylamide gel in TBE (80 mM Tris-borate, 1 mM EDTA) at 4°C for 16 h at 4 mA. The gels were stained with toluidine blue (0.05% [wt/vol] toluidine blue in 20% methanol, 2% glycerol) and scanned after destaining.

### Polysome profiling

Extracts were prepared from 100 mL cultures of fission yeast cells prior to (time 0) and after 4, 8, 12, and 24 h of inositol starvation and phosphate starvation. The cultures were adjusted to 100 µg/mL cycloheximide and incubated for 15 min at 30°C prior to placement on ice and harvesting the cells by centrifugation at 4°C. The cells were resuspended in 1 mL of ice-cold polysomal lysis buffer (PLB) (64). Cells were washed with PLB, re-pelleted, frozen on dry ice, and stored at −80°C. Cells were resuspended in 400 µL ice-cold PLB and lysed by four cycles of bead beating (6.5 m/s for 30 s, with 5 min incubation on ice between cycles), and extracts were prepared as described (64). 10%–50% sucrose gradients (11-mL) containing PLB were prepared using the BioComp Gradient Master. Aliquots of cell extracts, containing 18 *A*_260_ units in 400 µL of PLB, were applied to the chilled sucrose gradients, which were then centrifuged in a ThermoScientific TH-641 swinging bucket rotor at 37,000 rpm for 160 min at 4°C. The gradients were fractionated while continuously monitoring *A*_260_ by using the BioComp Gradient Station equipped with Triax software.

### Preparation of extracts for profiling of lipids and polar metabolites

Five independent cultures of fission yeast cells were maintained in logarithmic growth at 30°C in ePMGT(+INO) medium for 24 h, at which time cells (from cultures at *A*_600_ of 0.4 to 0.8) were harvested by centrifugation, washed with water, and resuspended at *A*_600_ of ~0.2 in ePMGT(+INO) or at *A*_600_ of ~0.1 in ePMGT(–INO). The cultures were incubated at 30°C and diluted as needed to maintain *A*_600_ below 0.8. Aliquots (3.15 *A*_600_ units) of cells grown for 24 h in ePMGT±INO were collected by vacuum filtration onto a 25 mm 0.2 µm nylon membrane (Millipore, Cat No. GNWP02500), and the membranes were immediately submerged in 1.1 mL of cold extraction solvent containing methanol:chloroform (1:2), 0.5 µM EquiSPLASH (Avanti, Cat No. 330731), and 1.5 µM ^13^C/^15^N amino acid mix (Cambridge Isotopes, Cat No. MSK-A2-1.2). The samples were vortexed and subjected to three cycles of freezing on dry ice and thawing at –20°C. One technical replicate for each independent culture and five media “blank” controls (ePMGT±INO) were collected by filtration and processed in parallel. Filters were removed using clean forceps, and 400 µL of ice-cold water was added to each sample. Samples were briefly vortexed and incubated on ice for 10 min. Subsequently, 200 µL of ice-cold methanol was added, and samples were centrifuged at 16,000 × *g* for 5 min at 4°C. For polar metabolite profiling, the upper aqueous phase was collected, transferred to 1.5 mL Eppendorf tubes, and dried using a vacuum evaporator (Genevac EZ-2 Elite) for 3 h. For lipid profiling, the lower chloroform phase was collected using a Hamilton syringe, transferred to glass vials, and dried under a gentle stream of nitrogen.

### Lipid profiling by liquid chromatography/mass spectrometry (LC-MS)

Dried lipid extracts were resuspended in 100 µL of 1:1 acetonitrile:isopropanol, vortexed, incubated at room temperature for 20 min, and centrifuged at 1,000 × g for 10 min at 25°C. Lipid analysis was performed using an Agilent 6546 Q-TOF mass spectrometer in both positive and negative ionization modes, coupled to a ZORBAX Eclipse Plus C18 column (100 mm × 2.1 mm, 1.8 µm particle size; Agilent Technologies). Mobile phase A consisted of 10 mM ammonium formate in 50:30:20 water:acetonitrile:isopropanol. Mobile phase B consisted of 10 mM ammonium formate in 1:9:90 water:acetonitrile:isopropanol. The LC gradient was as follows: 0 min, 0% B; 2.7 min, 45% B; 2.8 min, 53% B; 9 min, 65% B; 9.1 min, 89% B; 11 min, 92% B; 11.1 min, 100% B; 12 min, 10% B; 15 min, 10% B. The flow rate was 0.4 mL/min; column temperature was maintained at 60°C, and the injection volume was 5 µL. MS source parameters were as follows: gas temperature, 200°C; gas flow, 10 L/min; nebulizer pressure, 50 psig; sheath gas temperature, 300°C; sheath gas flow, 12 L/min; VCap, 3,000 V; nozzle voltage, 0 V; fragmentor, 150 V. Data were acquired from *m*/*z* 50–1,700 with active reference mass correction (positive mode: *m*/*z* 322.0481 and 922.0097; negative mode: *m*/*z* 301.9981 and 966.0007). Five iterative MS/MS acquisitions were performed on pooled lipid extracts in both polarities for lipid annotation. Lipid identification was performed using Agilent Lipid Annotator (v1.0.1) (65), and peak picking and integration were performed using Skyline (66).

### Polar metabolite profiling by LC-MS

Dried polar extracts were resuspended in 70 µL water for ion-pair chromatography or in 100 µL of 60:40 acetonitrile:water (for HILIC chromatography), vortexed, incubated on ice for 20 min, and centrifuged at 20,000 × *g* for 20 min at 4°C. Ion-pair chromatography was conducted using an XSelect HSS T3 column (150 mm × 2.1 mm, 3.5 µm; Waters) coupled to an Agilent 6230 TOF mass spectrometer with a Dual Jet Stream source in negative ion mode. Mobile phase A was composed of 5 mM octylamine and 5 mM acetic acid in water; Mobile phase B contained the same reagents in methanol. The gradient was 0–3.5 min, 1% B; 4 min, 35% B; 15 min, 35% B; 20 min, 100% B; 20.1–22.1 min, 100% B; 22.1–27 min, 1% B. The flow rate was 0.3 mL/min (0–20 min) and increased to 0.4 mL/min (20.1–27 min). A post-column flow of acetone:DMSO (90:10) at 0.3 mL/min was maintained throughout. Column temperature was 40°C; injection volume was 5 µL. Data were acquired from *m*/*z* 50–1,700 with active reference mass correction (*m*/*z*: 119.0363 and 966.0007). HILIC chromatography was performed using an Acquity UPLC BEH Amide column (150 mm × 2.1 mm, 1.7 µm; Waters) connected to an Agilent 6545 Q-TOF mass spectrometer with a Dual Jet Stream source in positive ion mode. Mobile phase A consisted of 10 mM ammonium acetate in 10% acetonitrile with 0.2% acetic acid, and Mobile phase B consisted of 10 mM ammonium acetate in 90% acetonitrile with 0.2% acetic acid. The LC gradient was 0–9 min, 95% B; 9–13 min, linear decrease to 30% B; 13–14 min, 30% B; 14.5–20 min, 95% B. The flow rate was 0.4 mL/min; column temperature was 40°C; injection volume was 5 µL. MS parameters were gas temperature, 300°C; gas flow, 10 L/min; nebulizer pressure, 35 psig; sheath gas temperature, 350°C; sheath gas flow, 12 L/min; VCap, 4,000 V; nozzle voltage, 1,000 V; fragmentor, 125 V. Data were acquired from *m*/*z* 50–1,700 with active reference mass correction (*m*/*z* 121.0508 and 922.0097). Peak identification and integration were carried out using Skyline (66).

## Data Availability

The RNA-seq data in this publication have been deposited in NCBI’s Gene Expression Omnibus and are accessible through GEO Series accession number GSE336424.

## SUPPLEMENTAL MATERIAL

Supplemental material mbio.01864-26-s0001.docx Figure S1 and legends to Tables S1 and S2.

Table S1 mbio.01864-26-s0002.xlsx Coding genes.

Table S2 mbio.01864-26-s0003.xlsx Biological processes.

ASM does not own the copyrights to Supplemental Material that may be linked to, or accessed through, an article. The authors have granted ASM a non-exclusive, world-wide license to publish the Supplemental Material files. Please contact the corresponding author directly for reuse.
