# Supplementary material for: Cellular adaptations of fission yeast to inositol starvation
Source: mBio. 2026 Sep 21;17(10):e01864-26. doi: 10.1128/mbio.01864-26 (PMC13712823; doi:10.1128/mbio.01864-26)
Supplement: Supplementary file 1 [file mbio.01864-26-s0001.docx]

Supplementary Material

**Cellular adaptations of fission yeast to inositol starvation**

Ana M. Sanchez, Jill Babor, Michelle Saoi, Isabel Prucker, Henning J. Jessen, Beate Schwer, and Stewart Shuman

Figure S1

Supplemental Tables S1, S2

[Tables S1 and S2 are .xls files]

| **Sample** | **Total Paired Reads** | **Mapped Reads** |
| --- | --- | --- |
| 0h (1) | 37,373,921 | 36,760,640 (98%) |
| 0h (2) | 40,497,063 | 39,878,064 (98%) |
| 0h (3) | 35,545,289 | 35,019,119 (98%) |
| 4h (1) | 35,803,662 | 35,253,879 (98%) |
| 4h (2) | 33,229,412 | 32,694,796 (98%) |
| 4h (3) | 35,289,224 | 34,770,821 (98%) |
| 8h (1) | 34,490,355 | 33,909,434 (98%) |
| 8h (2) | 31,639,396 | 31,119,034 (98%) |
| 8h (3) | 34,955,015 | 34,393,822 (98%) |
| 12h (1) | 35,589,829 | 34,956,414 (98%) |
| 12h (2) | 30,873,904 | 30,332,268 (98%) |
| 12h (3) | 32,145,850 | 31,641,759 (98%) |
| 24h (1) | 29,730,446 | 29,205,032 (98%) |
| 24h (2) | 30,178,822 | 29,629,625 (98%) |
| 24h (3) | 36,532,675 | 35,876,761 (98%) |

| **Sample pairs** | **Pearson Coefficient** |
| --- | --- |
| 0h (1) vs (2) | 0.991 |
| 0h (1) vs (3) | 0.989 |
| 0h (2) vs (3) | 0.990 |
| 4h (1) vs (2) | 0.990 |
| 4h (1) vs (3) | 0.988 |
| 4h (2) vs (3) | 0.989 |
| 8h (1) vs (2) | 0.989 |
| 8h (1) vs (3) | 0.987 |
| 8h (2) vs (3) | 0.986 |
| 12h (1) vs (2) | 0.989 |
| 12h (1) vs (3) | 0.986 |
| 12h (2) vs (3) | 0.987 |
| 24h (1) vs (2) | 0.986 |
| 24h (1) vs (3) | 0.980 |
| 24h (2) vs (3) | 0.981 |

Figure S1. RNA-seq read counts and data reproducibility for triplicate biological replicates.

Table S1: Coding genes that are UP or DOWN regulated in inositol starvation. The fold change in transcript level at the indicated times post-starvation is expressed as the log_2_ of the fold change compared to the “time 0” control.

Table S2: Impact of inositol starvation on selected biological processes.
